# Suicidal Thoughts and Behaviors Among High School Students — Youth Risk Behavior Survey, United States, 2021

**DOI:** 10.15585/mmwr.su7201a6

**Published:** 2023-04-28

**Authors:** Elizabeth M. Gaylor, Kathleen H. Krause, Laura E. Welder, Adina C. Cooper, Carmen Ashley, Karin A. Mack, Alexander E. Crosby, Eva Trinh, Asha Z. Ivey-Stephenson, Lisa Whittle

**Affiliations:** ^1^Division of Injury Prevention, National Center for Injury Prevention and Control, CDC; ^2^Division of Adolescent and School Health, National Center for HIV, Viral Hepatitis, STD, and TB Prevention, CDC; ^3^Department of Community Health & Preventive Medicine, Morehouse School of Medicine, Atlanta, Georgia

## Abstract

Suicide is the third leading cause of death among high school-aged youths aged 14–18 years. The 2021 suicide rate for this age group was 9.0 per 100,000 population. Updating a previous analysis of the Youth Risk Behavior Survey during 2009–2019, this report uses 2019 and 2021 data to examine high school students’ reports of suicidal thoughts and behaviors. Prevalence estimates are reported by grade, race and ethnicity, sexual identity, and sex of sexual contacts. Unadjusted logistic regression models were used to calculate prevalence differences comparing 2019 to 2021 and prevalence ratios comparing suicidal behavior between subgroups across demographic characteristics to a referent group. From 2019 to 2021, female students had an increased prevalence of seriously considered attempting suicide (from 24.1% to 30%), an increase in making a suicide plan (from 19.9% to 23.6%), and an increase in suicide attempts (from 11.0% to 13.3%). In addition, from 2019 to 2021, Black or African American (Black), Hispanic or Latino (Hispanic), and White female students had an increased prevalence of seriously considered attempting suicide. In 2021, Black female students had an increased prevalence of suicide attempts and Hispanic female students had an increased prevalence of suicide attempts that required medical treatment compared with White female students. Prevalence of suicidal thoughts and behaviors remained stable overall for male students from 2019 to 2021. A comprehensive approach to suicide prevention with a focus on health equity is needed to address these disparities and reduce prevalence of suicidal thoughts and behaviors for all youths. School and community-based strategies include creating safe and supportive environments, promoting connectedness, teaching coping and problem solving, and gatekeeper training.

## Introduction

Suicide presents a major challenge to public health in the United States and globally (*1*). In 2021, a total of 48,183 persons (all ages) died from suicide; suicide was the 11th leading cause of death overall in the United States, accounting for approximately 1.4% of all deaths (*2*). Although suicide and suicidal behaviors are a public health concern across the life span, youths aged 14–18 years present unique prevention challenges. Among high school-age youths aged 14–18 years, 1,952 suicides occurred in 2021, making suicide the third leading cause of death for this age group (2021 rate = 9.0 per 100,000 population). Suicide accounted for approximately one fifth of deaths (18.6%) among this age group (*2*). Deaths are only a portion of the burden of suicidal behavior; suicide attempts and suicidal thoughts among youths exceed deaths among this group. In 2020, according to data from a nationally representative sample of emergency departments (EDs), approximately 105,000 youths aged 14–18 years visited EDs for self-harm injuries (*3*).

One of the main objectives of *Healthy People 2030* Mental Health and Mental Disorders is to reduce suicide attempts by youths (*4*). The Youth Risk Behavior Survey (YRBS) monitors six categories of priority health behaviors and experiences among high school students with four questions related to suicide (*5*). This report summarizes 2021 YRBS data regarding suicidal thoughts and behaviors among high school students and compares results with 2019; this report also updates a previous analysis of the YRBS examining 2009–2019 data (*6*). The findings of this report indicate the need for a comprehensive public health approach to youth suicide prevention with attention to disproportionately affected populations.

## Methods

### Data Source

This report includes data from the 2019 (N = 13,677) and 2021 (N = 17,232) YRBS, a cross-sectional, school-based survey conducted biennially since 1991. Each survey year, CDC collects data from a nationally representative sample of public and private school students in grades 9–12 in the 50 U.S. states and the District of Columbia. Additional information about YRBS sampling, data collection, response rates, and processing is available in the overview report of this supplement (*5*). The prevalence estimates for suicidal thoughts and behaviors for the overall study population and by sex, race and ethnicity, grade, and sexual identity are available at https://nccd.cdc.gov/youthonline/App/Default.aspx. The full YRBS questionnaire, data sets, and documentation are available at https://www.cdc.gov/healthyyouth/data/yrbs/index.htm. This activity was reviewed by CDC and was conducted consistent with applicable federal law and CDC policy.*

### Measures

Four questions about suicidal thoughts and behavior are the focus of this report. The first asked, “During the past 12 months, did you ever seriously consider attempting suicide?”; the second, “During the past 12 months, did you make a plan about how you would attempt suicide?”; the third, “During the past 12 months, how many times did you actually attempt suicide?”; and the fourth, “If you attempted suicide during the past 12 months, did any attempt result in an injury, poisoning, or overdose that had to be treated by a doctor or nurse?” All questions had response options of yes or no except for the question about how many times a student attempted suicide, which was recoded to reflect whether a student had attempted suicide: yes (1 time, 2 or 3 times, 4 or 5 times, or 6 or more times) versus no (0 times). The question about needing medical treatment for a suicide attempt had an additional response option of “I did not attempt suicide during the past 12 months,” which was recoded to no.

Demographic variables included sex, measured as female or male and grade, measured as 9, 10, 11, or 12. Race and ethnicity was coded as a composite of two questions. First, students were asked, “Are you Hispanic or Latino?” This question measured as yes versus no; regardless of how students responded to the race question, those who answered yes were coded as Hispanic or Latino (Hispanic). Second, students were asked, “What is your race? Check all that apply” and coded as American Indian or Alaska Native (AI/AN), Asian, Black or African American (Black), Hispanic, Native Hawaiian or other Pacific Islander (NH/OPI), and White. Students who selected more than one race were coded as multiracial. (Persons of Hispanic origin might be of any race but were categorized as Hispanic; all racial groups were non-Hispanic.) Sexual orientation was measured by sexual identity and sex of sexual contacts. Sexual identity, measured as heterosexual; lesbian, gay, bisexual, questioning, or other (LGBQ+), had new response options in 2021 and was not comparable to the sexual identity measure from 2019. Sex of sexual contacts (“During your life, with whom have you had sexual contact?”) was measured as: “I have never had sexual contact,” “females,” “males,” or “females and males.” Responses were compared with the student’s sex to create the following categories: no sexual contact, opposite sex only (e.g., female students who have only ever had sexual contact with males), same sex only, or both sexes (e.g., female students who reported contact with females only or females and males).

### Analysis

Prevalence estimates and 95% CIs were calculated for each of the four suicidal measures, stratified by sex (historically, female youths are more likely to have suicidal thoughts and attempts, whereas male youths are more likely to die by suicide) (*6*), for the years 2019 and 2021. Sex-stratified prevalence estimates were further stratified by race and ethnicity, grade, sexual identity, and sex of sexual contacts. Using unadjusted logistic regression models with a statement to generate predicted marginal proportions, prevalence difference (PD) and prevalence ratios (PRs) were calculated to detect a difference in prevalence of a suicidal behavior for 2019 as compared with 2021 within a stratum (e.g., AI/AN female students). Using 2021 data only, PR were calculated to detect a difference in the prevalence of a suicidal behavior between subgroups across a demographic characteristic as compared with a referent group. A p value of <0.05 and 95% CI that did not cross the null value of 1.0 were used to determined statistical significance. The absolute value of the prevalence difference is presented. Prevalence estimates with a denominator <30 were considered statistically unreliable and therefore were suppressed (*5*). All analyses were conducted using SUDAAN (version 11.0.3; RTI International).

## Results

### Seriously Considered Attempting Suicide

Approximately one third (30.0%) of female students in 2021 reported that they had seriously considered attempting suicide during the 12 months before the survey, a substantial increase compared with 2019 (24.1%) (Table 1). The percentage of male students reporting that they had seriously considered attempting suicide were similar during the study period (2019 = 13.3%; 2021 = 14.3%). Increases in seriously considered attempting suicide differed by race, grade, and sex of sexual contacts among female students. For example, the prevalence significantly increased among Black (PD = 6.8%; PR = 1.29), Hispanic (PD = 6.0%; PR = 1.27), and White (PD = 7.1%; PR = 1.29) female students. Among male students, although the overall prevalence were similar, increases were observed among Hispanic (PD = 2.8%; PR = 1.24), 11th-grade (PD = 3.2%; PR = 1.23), opposite sex only sexual contacts (PD = 4.0%; PR = 1.27), and same sex or both sex sexual contacts (PD = 18.2%; PR = 1.47).

**TABLE 1:** Prevalence of seriously considered attempting suicide, prevalence difference, prevalence ratio comparing 2019 with 2021, and 2021 prevalence ratio by demographic characteristics — Youth Risk Behavior Survey, United States, 2019 and 2021

Characteristic	2019* % (95% CI)	2021* % (95% CI)	From 2019 to 2021 PD^†^	From 2019 to 2021 PR (95% CI)	2021 PR (95% CI)
**Female**						
**Total**	**24.1 (22.3–26.0)**	**30.0 (28.5–31.4)**	**5.9^§^**	**1.24 (1.14–1.36)^¶^**	**N/A**
Race and ethnicity^**^						
American Indian or Alaska Native	36.6 (20.7–56.1)	36.8 (25.3–50.0)	0.2	1.01 (0.55–1.83)	1.17 (0.81–1.70)
Asian	22.0 (16.9–28.1)	24.2 (19.8–29.1)	2.2	1.10 (0.80–1.51)	0.77 (0.63–0.94)**^¶^**
Black or African American	23.7 (20.7–27.1)	30.5 (25.6–36.0)	6.8^§^	1.29 (1.04–1.59)**^¶^**	0.97 (0.79–1.20)
Native Hawaiian or other Pacific Islander	—^††^	25.3 (7.6–58.3)	—	—	0.80 (0.28–2.31)
White	24.3 (21.9–26.9)	31.4 (29.2–33.7)	7.1^§^	1.29 (1.14–1.46)^¶^	Ref
Hispanic or Latino	22.7 (19.3–26.5)	28.7 (26.8–30.8)	6.0^§^	1.27 (1.07–1.50)^¶^	0.92 (0.83–1.01)
Multiracial	33.1 (27.8–38.7)	28.5 (22.7–35.2)	4.6	0.86 (0.66–1.13)	0.91 (0.71–1.16)
**Grade**						
9	23.7 (20.7–27.0)	30.7 (27.4–34.3)	7.0^§^	1.30 (1.09–1.54)^¶^	1.20 (1.05–1.37)**^¶^**
10	23.6 (20.3–27.3)	33.6 (30.7–36.7)	10.0^§^	1.42 (1.20–1.69)**^¶^**	1.31 (1.16–1.48)**^¶^**
11	24.9 (22.5–27.6)	29.7 (26.8–32.8)	4.8^§^	1.19 (1.04–1.37)**^¶^**	1.16 (1.04–1.30)**^¶^**
12	24.0 (20.7–27.6)	25.6 (23.8–27.4)	1.6	1.07 (0.91–1.25)	Ref
**Sexual identity**						
Heterosexual	N/A	19.9 (18.5–21.5)	N/A	N/A	Ref
Lesbian or gay	N/A	41.0 (31.8–50.9)	N/A	N/A	2.06 (1.59–2.66)^¶^
Bisexual	N/A	51.9 (47.6–56.1)	N/A	N/A	2.60 (2.32–2.91)^¶^
Questioning	N/A	36.0 (30.4–41.9)	N/A	N/A	1.80 (1.51–2.15)^¶^
Other	N/A	47.9 (43.6–52.1)	N/A	N/A	2.40 (2.18–2.64)^¶^
**Sex of sexual contacts**						
Opposite sex only	25.3 (22.8–28.0)	35.3 (32.4–38.3)	10.0^§^	1.40 (1.23–1.59)^¶^	1.68 (1.52–1.85)^¶^
Same sex only or both sexes	59.2 (52.5–65.6)	58.4 (53.0–63.7)	0.8	0.99 (0.86–1.14)	2.78 (2.51–3.08)^¶^
No sexual contact	16.1 (14.2–18.3)	21.0 (19.8–22.4)	4.9^§^	1.30 (1.13–1.50)^¶^	Ref
**Male**						
**Total**	**13.3 (12.2–14.5)**	**14.3 (13.3–15.4)**	**1.0**	**1.08 (0.96–1.20)**	N/A
Race and ethnicity**						
American Indian or Alaska Native	32.8 (17.9–52.2)	19.1 (9.7–34.0)	13.7	0.58 (0.26–1.32)	1.32 (0.71–2.44)
Asian	17.3 (12.6–23.3)	11.8 (8.9–15.4)	5.5	0.68 (0.46–1.02)	0.81 (0.61–1.09)
Black or African American	10.7 (8.2–13.7)	13.0 (10.4–16.0)	2.3	1.22 (0.88–1.69)	0.89 (0.71–1.12)
Native Hawaiian or other Pacific Islander	7.7 (1.8–28.1)	18.7 (8.3–36.9)	11.0	2.42 (0.50–11.77)	1.29 (0.60–2.78)
White	13.8 (12.3–15.3)	14.5 (13.1–16.0)	0.8	1.05 (0.91–1.22)	Ref
Hispanic or Latino	11.4 (9.8–13.3)	14.2 (12.1–16.6)	2.8^§^	1.24 (1.00–1.54)^¶^	0.98 (0.82–1.17)
Multiracial	17.5 (12.4–24.1)	18.1 (14.5–22.4)	0.6	1.04 (0.70–1.53)	1.25 (0.99–1.58)
**Grade**						
9	11.9 (9.9–14.2)	11.9 (9.8–14.5)	0.0	1.00 (0.77–1.30)	0.72 (0.58–0.90)^¶^
10	13.2 (11.1–15.8)	12.7 (11.2–14.4)	0.5	0.96 (0.77–1.19)	0.77 (0.64–0.93)^¶^
11	13.6 (11.5–16.0)	16.8 (15.0–18.7)	3.2^§^	1.23 (1.02–1.50)^¶^	1.02 (0.84–1.24)
12	14.9 (12.4–17.7)	16.5 (14.2–19.0)	1.6	1.11 (0.88–1.39)	Ref
**Sexual identity**						
Heterosexual	N/A	11.6 (10.6–12.8)	N/A	N/A	Ref
Lesbian or gay	N/A	35.5 (27.7–44.1)	N/A	N/A	3.05 (2.44–3.82)^¶^
Bisexual	N/A	40.2 (31.9–49.1)	N/A	N/A	3.45 (2.81–4.25)^¶^
Questioning	N/A	28.0 (21.1–36.1)	N/A	N/A	2.40 (1.79–3.24)^¶^
Other	N/A	45.8 (29.1–63.5)	N/A	N/A	3.93 (2.58–5.99)^¶^
**Sex of sexual contacts**						
Opposite sex only	14.6 (12.9–16.5)	18.6 (16.7–20.5)	4.0^§^	1.27 (1.08–1.49)^¶^	2.13 (1.79–2.54)^¶^
Same sex only or both sexes	39.1 (29.3–49.9)	57.3 (49.5–64.8)	18.2^§^	1.47 (1.09–1.97)^¶^	6.58 (5.46–7.94)^¶^
No sexual contact	9.7 (8.1–11.7)	8.7 (7.6–10.0)	1.0	0.89 (0.71–1.12)	Ref

**Abbreviations:** N/A = not applicable; PD = prevalence difference; PR = prevalence ratio; Ref = referent group.

* 2019: N = 13,677 respondents; 2021: N=17,232 respondents. Because the state and local questionnaires differ by jurisdiction, students in these schools were not asked all national YRBS questions. Therefore, the total number (N) of students answering each question varied. Percentages in each category are calculated on the known data.

^†^ Absolute value presented.

^§^ On the basis of *t*-test with Taylor series linearization (p<0.05).

^¶^ 95% CI did not cross the null value of 1.0.

** Persons of Hispanic origin might be of any race but were categorized as Hispanic; all racial groups were non-Hispanic.

^††^ Prevalence estimates with a denominator <30 were considered statistically unreliable and therefore were suppressed.

In 2021, Asian female students had a lower prevalence of seriously considered attempting suicide compared with White female students (PR = 0.77). The prevalence of female students in 9th, 10th, and 11th grade who seriously considered attempting suicide was significantly greater (PR = 1.20, 1.31, and 1.16, respectively) than female students in 12th grade. In addition, prevalence was significantly higher among LGBQ+ female students (PR = 2.06 lesbian or gay; 2.60 bisexual; 1.80 questioning; 2.40 other) compared with heterosexual students. Among males in 2021, students in 9th and 10th grade were less likely (PR = 0.72 and 0.77, respectively) than students in 12th grade to report seriously considered attempting suicide. Similar to female students, prevalence was significantly higher among LGBQ+ male students (PR = 3.05 gay; 3.45 bisexual; 2.40 questioning; 3.93 other) compared with heterosexual students.

### Made a Suicide Plan

Approximately one fourth (23.6%) of female students in 2021 reported making a suicide plan during the 12 months before the survey, a significant increase over 2019 (19.9%) (Table 2). The percentage of male students reporting making a suicide plan was stable during the study period (2019 = 11.3%; 2021 = 11.6%). Significant increases in prevalence between 2019 and 2021 of reporting having made a suicide plan were observed among Hispanic (PD = 5.2%; PR = 1.26), White (PD = 3.7%; PR = 1.19), 9th grade (PD = 4.7%; PR = 1.23), and 10th grade (PD = 6.6%; PR = 1.33) female students as well as female students who had sex only with opposite sex partners (PD = 7.2%; PR = 1.35). NH/OPI male students (PD = 12.8%), male students who had sexual contact with opposite sex partners only (PD = 3.2%; PR = 1.25), and those who had sexual contact with same sex or both sex partners (PD = 15.5%; PR = 1.50) reported significant increases in the prevalence of having made a suicide plan from 2019 to 2021.

**TABLE 2:** Prevalence of made a suicide plan, prevalence difference and prevalence ratio comparing 2019 with 2021, and 2021 prevalence ratio by demographic characteristics — Youth Risk Behavior Survey, United States, 2019 and 2021

Characteristic	2019* % (95% CI)	2021* % (95% CI)	From 2019 to 2021 PD^†^	From 2019 to 2021 PR (95% CI)	2021 PR (95% CI)
**Female**						
**Total**	**19.9 (18.4–21.6)**	**23.6 (22.1–25.1)**	**3.7** ^§^	**1.18 (1.07**–**1.31)^¶^**	**N/A**
Race and ethnicity**						
American Indian or Alaska Native	29.8 (17.4–46.1)	27.0 (16.4–41.1)	2.7	0.91 (0.47–1.77)	1.18 (0.72–1.94)
Asian	19.2 (15.2–24.0)	22.9 (19.5–26.6)	3.6	1.19 (0.91–1.56)	1.00 (0.82–1.21)
Black or African American	20.4 (17.6–23.5)	24.3 (20.7–28.2)	3.9	1.19 (0.97–1.47)	1.06 (0.88–1.28)
Native Hawaiian or other Pacific Islander	—^††^	23.3 (8.9–48.5)	—	—	1.02 (0.42–2.43)
White	19.2 (16.9–21.8)	22.9 (20.7–25.3)	3.7^§^	1.19 (1.02–1.40)^¶^	Ref
Hispanic or Latino	19.6 (16.9–22.6)	24.8(22.6–27.0)	5.2^§^	1.26 (1.07–1.49)^¶^	1.08 (0.95–1.23)
Multiracial	28.2 (23.4–33.6)	24.4 (19.3–30.5)	3.8	0.87 (0.65–1.15)	1.07 (0.82–1.38)
**Grade**						
9	20.4 (17.9–23.2)	25.1 (22.3–28.3)	4.7^§^	1.23 (1.03–1.46)^¶^	1.28 (1.11–1.48)^¶^
10	20.3 (17.2–23.7)	26.9 (24.8–29.1)	6.6^§^	1.33 (1.11–1.58)^¶^	1.37 (1.24–1.52)^¶^
11	20.4 (17.6–23.5)	22.4 (19.2–26.0)	2.0	1.10 (0.89–1.35)	1.14 (0.94–1.38)
12	18.5 (15.7–21.6)	19.6 (17.9–21.4)	1.1	1.06 (0.89–1.27)	Ref
**Sexual identity**						
Heterosexual	N/A	15.1 (13.7–16.6)	N/A	N/A	Ref
Lesbian or gay	N/A	31.1 (22.1–41.8)	N/A	N/A	2.06 (1.47–2.88)^¶^
Bisexual	N/A	43.2 (39.1–47.5)	N/A	N/A	2.87 (2.53–3.24)^¶^
Questioning	N/A	28.8 (23.9–34.3)	N/A	N/A	1.91 (1.57–2.32)^¶^
Other	N/A	36.8 (32.3–41.4)	N/A	N/A	2.44 (2.13–2.79)^¶^
**Sex of sexual contacts**						
Opposite sex only	20.7 (18.4–23.3)	27.9 (25.4–30.7)	7.2^§^	1.35 (1.16–1.57)^¶^	1.81 (1.54–2.13)^¶^
Same sex only or both sexes	48.2 (42.8–53.6)	51.3 (46.3–56.2)	3.1	1.06 (0.92–1.23)	3.33 (2.84–3.90)^¶^
No sexual contact	13.8 (12.3–15.6)	15.4 (13.8–17.2)	1.6	1.11 (0.95–1.31)	Ref
**Male**						
**Total**	**11.3 (10.3–12.4)**	**11.6 (10.5–12.8)**	**0.3**	**1.03 (0.90–1.17)**	**N/A**
Race and ethnicity**						
American Indian or Alaska Native	19.4 (7.6–41.4)	17.1 (8.0–32.8)	2.3	0.88 (0.29–2.66)	1.53 (0.78–2.99)
Asian	13.0 (8.7–19.2)	10.3 (6.5–15.8)	2.8	0.79 (0.44–1.41)	0.91 (0.56–1.50)
Black or African American	10.1 (7.3–13.9)	11.3 (8.7–14.5)	1.2	1.12 (0.75–1.68)	1.01 (0.75–1.35)
Native Hawaiian or other Pacific Islander	5.3 (1.1–22.5)	18.1 (11.8–26.8)	12.8^§^	3.41 (0.70–16.58)	1.62 (1.04–2.50)^¶^
White	12.0 (10.6–13.5)	11.2 (9.8–12.8)	0.8	0.94 (0.78–1.12)	Ref
Hispanic or Latino	9.6 (8.0–11.4)	11.9 (9.9–14.3)	2.3	1.25 (0.97–1.60)	1.06 (0.86–1.30)
Multiracial	15.8 (11.5–21.1)	15.0 (10.9–20.4)	0.7	0.95 (0.62–1.47)	1.34 (0.93–1.93)
**Grade**						
9	9.5 (7.9–11.4)	11.4 (9.3–14.0)	1.9	1.20 (0.91–1.58)	0.98 (0.81–1.18)
10	10.4 (8.6–12.4)	9.8 (8.0–12.0)	0.5	0.95 (0.73–1.24)	0.84 (0.63–1.13)
11	12.1 (10.3–14.2)	13.7 (12.1–15.5)	1.6	1.13 (0.93–1.38)	1.17 (0.95–1.45)
12	13.6 (11.4–16.1)	11.7 (9.9–13.8)	1.9	0.86 (0.68–1.09)	Ref
**Sexual identity**						
Heterosexual	N/A	9.5 (8.5–10.6)	N/A	N/A	Ref
Lesbian or gay	N/A	34.1 (24.9–44.6)	N/A	N/A	3.59 (2.69–4.80)^¶^
Bisexual	N/A	30.6 (23.7–38.6)	N/A	N/A	3.23 (2.50–4.16)^¶^
Questioning	N/A	24.5 (17.4–33.4)	N/A	N/A	2.58 (1.84–3.63)^¶^
Other	N/A	37.9 (23.8–54.3)	N/A	N/A	4.00 (2.60–6.15)^¶^
**Sex of sexual contacts**						
Opposite sex only	12.9 (11.5–14.6)	16.2 (14.4–18.1)	3.2^§^	1.25 (1.06–1.47)^¶^	2.38 (1.81–3.13)^¶^
Same sex only or both sexes	31.2 (23.8–39.7)	46.7 (37.6–56.1)	15.5^§^	1.50 (1.09–2.07)^¶^	6.89 (5.04–9.43)^¶^
No sexual contact	7.9 (6.7–9.4)	6.8 (5.4–8.5)	1.1	0.86 (0.64–1.14)	Ref

**Abbreviations:** N/A = not applicable; PD = prevalence difference; PR = prevalence ratio; Ref = referent group.

* 2019: N = 13,677 respondents; 2021: N=17,232 respondents. Because the state and local questionnaires differ by jurisdiction, students in these schools were not asked all national YRBS questions. Therefore, the total number (N) of students answering each question varied. Percentages in each category are calculated on the known data.

^†^ Absolute value presented.

^§^ On the basis of *t*-test with Taylor series linearization (p<0.05).

^¶^ Prevalence estimates with a denominator <30 were considered statistically unreliable and therefore were suppressed.

** Persons of Hispanic origin might be of any race but were categorized as Hispanic; all racial groups were non-Hispanic.

^¶^ 95% CI did not cross the null value of 1.0.

In 2021, female students in 9th and 10th grade were significantly more likely (PR = 1.28 and 1.37, respectively) than 12th grade students to report having made a suicide plan. Female students reporting opposite sex only sexual contacts (PR = 1.81) and those with same sex or both sex partners were more likely (PR = 3.33) than those with no sexual contact to report having made a suicide plan. In 2021, NH/OPI male students (PR = 1.62) were significantly more likely than White male students to have made a suicide plan. Additionally, male students reporting opposite sex only sexual contacts (PR = 2.38) and those with same sex or both sex partners were more likely (PR = 6.89) than those with no sexual contact to report having made a suicide plan. Prevalence of having made a suicide plan was significantly higher among LGBQ+ students (females: PR = 2.06 lesbian or gay; 2.87 bisexual; 1.91 questioning; 2.44 other; males: PR = 3.59 gay; 3.23 bisexual; 2.58 questioning; 4.00 other) compared with heterosexual students.

### Attempted Suicide

Reports of suicide attempts during the 12 months before the survey significantly increased among female students (PD = 2.3%; PR = 1.21) and was unchanged among male students from 2019 to 2021 (Table 3). The prevalence of reported attempted suicide was 13.3% among females in 2021 and 6.6% among males. Increases in reports of suicide attempts occurred among White (PD = 3.0%; PR = 1.32), 10th grade (PD = 4.6%; PR = 1.41) female students, as well as among female students with opposite sex only sexual contacts (PD = 4.7%, PR = 1.41). In 2021, Black female students were more likely (PR = 1.43) than White female students to report having attempted suicide, as well as 9th and 10th grade (PR = 1.54 and 1.52 respectively) female students compared with 12th grade, and LGBQ+ students (PR = 1.86 lesbian or gay; 3.26 bisexual; 1.53 questioning; 2.47 other) compared with heterosexual students. Also in 2021, female students reporting opposite sex only sexual contacts (PR = 2.50) and those with same sex only or both sex partners (PR = 5.19) were more likely than those with no sexual contact to report having attempted suicide. In 2021, AI/AN and Black male students reports of attempted suicide were significantly higher (PR = 2.37, 2.05 respectively) than White male students. Among males in 2021, LGBQ+ students (PR = 3.93 gay; 3.44, bisexual; 2.81 questioning; 6.60 other) were more likely to have reported attempting suicide compared with heterosexual students, and male students reporting opposite sex only sexual contacts (PR = 2.96) and those with same sex only or both sex partners were more likely (PR = 10.72) than those with no sexual contact to report having attempted suicide.

**TABLE 3:** Prevalence of attempted suicide, prevalence difference and prevalence ratio comparing 2019 with 2021, and prevalence ratio by demographic characteristics in 2021 — Youth Risk Behavior Survey, United States, 2019 and 2021

Characteristic	2019* % (95% CI)	2021* % (95% CI)	From 2019 to 2021 PD^†^	From 2019 to 2021 PR (95% CI)	2021 PR (95% CI)
**Female**						
**Total**	**11.0 (9.7–12.5)**	**13.3 (12.0–14.7)**	**2.3** ^§^	**1.21 (1.03–1.41)^¶^**	**N/A**
Race and ethnicity**						
American Indian or Alaska Native	13.4 (5.3–30.1)	19.4 (11.3–31.3)	6.0	1.44 (0.53–3.94)	1.56 (0.87–2.79)
Asian	8.4 (4.8–14.2)	8.3 (4.9–13.8)	0.1	0.99 (0.47–2.07)	0.67 (0.37–1.19)
Black or African American	15.2 (10.8–20.9)	17.8 (14.1–22.3)	2.7	1.17 (0.79–1.74)	1.43 (1.10–1.86)^¶^
Native Hawaiian or other Pacific Islander	—^††^	14.4 (6.4–29.3)	—	—	1.16 (0.54–2.50)
White	9.4 (7.8–11.3)	12.4 (10.7–14.5)	3.0^§^	1.32 (1.04–1.67)^¶^	Ref
Hispanic or Latino	11.9 (9.0–15.6)	13.8 (12.0–15.9)	1.9	1.16 (0.85–1.57)	1.11 (0.90–1.37)
Multiracial	17.8 (13.1–23.7)	13.9 (10.6–18.0)	3.9	0.78 (0.53–1.16)	1.12 (0.80–1.56)
**Grade**						
9	12.8 (10.7–15.3)	15.8 (13.8–18.0)	3.0	1.23 (0.99–1.54)	1.54 (1.27–1.87)^¶^
10	11.0 (9.1–13.3)	15.6 (13.3–18.2)	4.6^§^	1.41 (1.11–1.81)^¶^	1.52 (1.21–1.91)^¶^
11	10.4 (8.1–13.3)	11.2 (8.8–14.1)	0.7	1.07 (0.76–1.50)	1.09 (0.86–1.38)
12	9.4 (6.9–12.6)	10.3 (8.7–12.1)	0.9	1.09 (0.78–1.53)	Ref
**Sexual identity**						
Heterosexual	N/A	8.1 (7.3–9.1)	N/A	N/A	Ref
Lesbian or gay	N/A	15.2 (10.2–21.9)	N/A	N/A	1.86 (1.27–2.73)^¶^
Bisexual	N/A	26.5 (23.2–30.2)	N/A	N/A	3.26 (2.73–3.89)^¶^
Questioning	N/A	12.5 (9.6–16.0)	N/A	N/A	1.53 (1.22–1.92)^¶^
Other	N/A	20.1 (15.9–25.1)	N/A	N/A	2.47 (1.95–3.12)^¶^
**Sex of sexual contacts**						
Opposite sex only	11.4 (9.5–13.5)	16.1 (14.1–18.4)	4.7^§^	1.41 (1.14–1.76)^¶^	2.50 (2.02–3.09)^¶^
Same sex only or both sexes	31.4 (27.0–36.1)	33.5 (29.3–38.0)	2.1	1.07 (0.88–1.29)	5.19 (4.16–6.48)^¶^
No sexual contact	6.1 (4.8–7.8)	6.4 (5.4–7.6)	0.3	1.06 (0.79–1.42)	Ref
**Male**						
**Total**	**6.6 (5.5–8.1)**	**6.6 (5.8–7.5)**	**0.0**	**1.00 (0.79–1.25)**	**N/A**
**Race and ethnicity^††^**						
American Indian or Alaska Native	34.3 (16.5–57.9)	13.0 (6.6–23.8)	21.3	0.38 (0.16–0.92)^¶^	2.37 (1.22–4.61)^¶^
Asian	7.1 (3.6–13.7)	4.7 (2.3–9.4)	2.4	0.66 (0.25–1.73)	0.86 (0.45–1.64)
Black or African American	8.5 (5.6–12.9)	11.2 (8.4–14.7)	2.7	1.31 (0.80–2.16)	2.05 (1.43–2.95)^¶^
Native Hawaiian or other Pacific Islander	—	6.1 (1.2–26.5)	—	—	1.11 (0.24–5.21)
White	6.4 (5.1–7.8)	5.5 (4.5–6.7)	0.9	0.86 (0.64–1.15)	Ref
Hispanic or Latino	5.5 (3.9–7.6)	6.5 (4.3–9.8)	1.1	1.20 (0.71–2.01)	1.20 (0.74–1.93)
Multiracial	7.3 (3.4–15.1)	8.1 (5.4–12.0)	0.8	1.11 (0.48–2.57)	1.48 (0.92–2.38)
**Grade**						
9	6.0 (4.5–7.9)	6.9 (5.4–8.8)	1.0	1.17 (0.81–1.68)	1.01 (0.79–1.31)
10	6.5 (4.7–9.0)	6.2 (5.0–7.6)	0.3	0.95 (0.64–1.39)	0.90 (0.76–1.08)
11	6.7 (5.2–8.8)	6.2 (4.6–8.3)	0.5	0.92 (0.62–1.36)	0.91 (0.64–1.28)
12	7.3 (5.2–10.0)	6.9 (5.8–8.0)	0.4	0.94 (0.66–1.35)	Ref
**Sexual identity**						
Heterosexual	N/A	5.0 (4.1–6.0)	N/A	N/A	Ref
Lesbian or gay	N/A	19.6 (12.5–29.3)	N/A	N/A	3.93 (2.58–5.99)^¶^
Bisexual	N/A	17.2 (12.2–23.6)	N/A	N/A	3.44 (2.32–5.11)^¶^
Questioning	N/A	14.0 (7.8–23.8)	N/A	N/A	2.81 (1.48–5.35)^¶^
Other	N/A	32.9 (20.7–47.9)	N/A	N/A	6.60 (3.94–11.05)^§^
**Sex of sexual contacts**						
Opposite sex only	7.5(5.8–9.6)	8.5 (7.2–10.0)	1.0	1.14 (0.84–1.53)	2.96 (2.27–3.86)^¶^
Same sex only or both sexes	26.5 (17.5–38.0)	30.8 (21.0–42.7)	4.3	1.16 (0.69–1.95)	10.72 (6.88–16.71)^¶^
No sexual contact	3.5 (2.6–4.8)	2.9 (2.2–3.7)	0.7	0.81 (0.55–1.19)	Ref

**Abbreviations:** N/A = not applicable; PD = prevalence difference; PR = prevalence ratio; Ref = referent group.

* 2019: N = 13,677 respondents; 2021: N=17,232 respondents. Because the state and local questionnaires differ by jurisdiction, students in these schools were not asked all national YRBS questions. Therefore, the total number (N) of students answering each question varied. Percentages in each category are calculated on the known data.

^†^ Absolute value presented.

^§^ On the basis of *t*-test with Taylor series linearization (p<0.05).

^¶^ 95% CI did not cross the null value of 1.0.

** Prevalence estimates with a denominator <30 were considered statistically unreliable and therefore were suppressed.

^††^ Persons of Hispanic origin might be of any race but were categorized as Hispanic; all racial groups were non-Hispanic.

### Attempted Suicide that Required Medical Treatment

The prevalence of attempted suicide that required medical treatment was relatively stable between 2019 and 2021 for female (2019 = 3.3%; 2021 = 3.9%) and male (2019 = 1.7%; 2021 = 1.7%) students overall and by student characteristics (Table 4). In 2021, female Hispanic students were more likely (PR = 1.31) than female White students, and 9th grade female students (PR = 1.51) were more likely than 12th grade students, to report an attempted suicide that required medical treatment. Bisexual and other identifying female students were more likely (PR = 4.23 and 3.04, respectively) than heterosexual female students to report an attempted suicide that required medical treatment. Black (PR = 2.64) and Hispanic (PR = 1.61) male students were more likely than White male students to report an attempted suicide that required medical treatment. Among males in 2021, LGBQ+ students (PR = 7.93 gay; 7.23 bisexual; 7.06 questioning; 14.31 other) were more likely to report an attempted suicide that required medical treatment compared with heterosexual students. Students with opposite sex only sexual contacts (PR = 5.08 female; 4.09 male) and those with same sex or both sexes sexual contacts were more likely (PR = 14.36 female; 30.15 male) than students with no sexual contact to report an attempted suicide that required medical treatment.

**TABLE 4:** Prevalence of attempted suicide that required medical treatment, prevalence difference and prevalence ratio comparing 2019 with 2021, and prevalence ratio by demographic characteristics in 2021 — Youth Risk Behavior Survey, United States, 2019 and 2021

Characteristic	2019* % (95% CI)	2021* % (95% CI)	From 2019 to 2021 PD^†^	From 2019 to 2021 PR (95% CI)	2021 PR (95% CI)
**Female**						
**Total**	**3.3 (2.6–4.2)**	**3.9 (3.1–4.8)**	**0.5**	**1.16 (0.84–1.60)**	**N/A**
**Race and ethnicity^¶^**						
American Indian or Alaska Native	—	1.0 (0.1–7.2)	—	—	—
Asian	1.9 (0.6–5.8)	2.2 (0.9–5.2)	0.3	1.13 (0.28–4.64)	0.61 (0.22–1.72)
Black or African American	3.8 (2.3–6.2)	5.5 (3.5–8.7)	1.7	1.46 (0.75–2.82)	1.56 (1.00–2.42)
Native Hawaiian or other Pacific Islander	—	1.3 (0.2–6.7)	—	—	—
White	2.9 (1.9–4.4)	3.5 (2.8–4.5)	0.6	1.22 (0.76–1.96)	Ref
Hispanic or Latino	3.6 (2.6–4.9)	4.7 (3.6–6.0)	1.1	1.30 (0.87–1.95)	1.31 (1.01–1.72)**
Multiracial	6.7 (3.2–13.2)	3.0 (1.5–5.9)	3.7	0.45 (0.17–1.19)	0.84 (0.48–1.48)
**Grade**						
9	3.3 (2.3–4.8)	4.8 (3.8–6.0)	1.5	1.44 (0.93–2.23)	1.51 (1.01–2.27)**
10	3.6 (2.3–5.5)	4.1 (2.9–5.8)	0.5	1.14 (0.66–1.96)	1.29 (0.88–1.90)
11	2.7 (1.7–4.3)	3.3 (2.4–4.6)	0.6	1.24 (0.70–2.17)	1.05 (0.67–1.64)
12	3.4 (2.2–5.3)	3.2 (2.1–4.8)	0.2	0.94 (0.51–1.72)	Ref
**Sexual identity**						
Heterosexual	N/A	2.1 (1.5–2.9)	N/A	N/A	Ref
Lesbian or gay	N/A	3.0 (1.4–6.3)	N/A	N/A	1.45 (0.63–3.31)
Bisexual	N/A	8.8 (6.3–12.1)	N/A	N/A	4.23 (2.89–6.17)**
Questioning	N/A	3.4 (1.9–6.0)	N/A	N/A	1.63 (0.88–3.02)
Other	N/A	6.3 (3.7–10.5)	N/A	N/A	3.04 (1.51–6.09)**
**Sex of sexual contacts**						
Opposite sex only	3.4 (2.4–4.8)	4.9 (3.7–6.4)	1.5	1.43 (0.92–2.23)	5.08 (2.99–8.63)**
Same sex only or both sexes	10.4 (7.5–14.2)	13.7 (10.8–17.3)	3.4	1.32 (0.89–1.96)	14.36 (9.00–22.91)**
No sexual contact	1.4 (0.8–2.4)	1.0 (0.6–1.6)	0.4	0.68 (0.33–1.41)	Ref
**Male**						
**Total**	**1.7 (1.3–2.3)**	**1.7 (1.4–2.0)**	**0.1**	**0.97 (0.70–1.34)**	**N/A**
**Race and ethnicity^¶^**						
American Indian or Alaska Native	16.0 (4.7–42.4)	—	—	—	—
Asian	1.4 (0.2–10.2)	1.1 (0.3–3.4)	0.3	0.76 (0.08–7.71)	0.87 (0.21–3.59)
Black or African American	2.9 (1.5–5.5)	3.3 (1.9–5.6)	0.4	1.14 (0.49–2.66)	2.64 (1.37–5.10)**
Native Hawaiian or other Pacific Islander	—	5.5 (0.9–27.8)	—	—	—
White	1.2 (0.8–1.9)	1.2 (0.9–1.8)	0.0	1.01 (0.57–1.77)	Ref
Hispanic or Latino	2.3 (1.4–3.9)	2.0 (1.5–2.7)	0.3	0.86 (0.48–1.54)	1.61 (1.02–2.55)**
Multiracial	0.9 (0.2–3.8)	3.1 (1.3–7.0)	2.2	3.32 (0.66–16.65)	2.50 (0.91–6.83)
**Grade**						
9	1.3 (0.7–2.3)	1.1 (0.6–2.4)	0.1	0.91 (0.36–2.30)	0.78 (0.35–1.71)
10	1.7 (0.9–3.3)	1.8 (0.9–3.4)	0.1	1.06 (0.43–2.61)	1.22 (0.47–3.20)
11	2.0 (1.2–3.2)	2.3 (1.4–3.7)	0.3	1.16 (0.60–2.26)	1.55 (0.77–3.12)
12	1.9 (1.0–3.9)	1.5 (0.9–2.3)	0.5	0.77 (0.34–1.74)	Ref
**Sexual identity**						
Heterosexual	N/A	0.9 (0.7–1.2)	N/A	N/A	Ref
Lesbian or gay	N/A	7.4 (3.2–16.1)	N/A	N/A	7.93 (3.75–16.77)**
Bisexual	N/A	6.8 (2.8–15.3)	N/A	N/A	7.23 (2.94–17.76)**
Questioning	N/A	6.6 (2.1–18.7)	N/A	N/A	7.06 (2.06–24.22)**
Other	N/A	13.4 (4.9–31.9)	N/A	N/A	14.31 (4.94–41.51)**
**Sex of sexual contacts**						
Opposite sex only	1.9 (1.3–2.9)	2.1 (1.6–2.8)	0.2	1.12 (0.69–1.81)	4.09 (2.23–7.48)**
Same sex only or both sexes	9.4 (4.9–17.6)	15.8 (10.4–23.2)	6.3	1.67 (0.79–3.54)	30.15 (14.99–60.64)**
No sexual contact	0.5 (0.3–1.1)	0.5 (0.3–0.9)	0.0	0.99 (0.42–2.33)	Ref

**Abbreviations:** N/A = not applicable; PD = prevalence difference; PR = prevalence ratio; Ref = referent group.

* 2019: N = 13,677 respondents; 2021: N=17,232 respondents. Because the state and local questionnaires differ by jurisdiction, students in these schools were not asked all national YRBS questions. Therefore, the total number (N) of students answering each question varied. Percentages in each category are calculated on the known data.

^†^ Absolute value presented.

^§^ Prevalence estimates with a denominator <30 were considered statistically unreliable and therefore were suppressed.

^¶^ Persons of Hispanic origin might be of any race but were categorized as Hispanic; all racial groups were non-Hispanic.

** 95% CI did not cross the null value of 1.0.

## Discussion

Overall results from the 2019 and 2021 YRBSs highlight stable prevalence of suicidal thoughts and behaviors among male students across all outcomes, with increases observed among certain subgroups of male students and significant increases among female students in three of four outcomes (i.e., suicidal thoughts, plans, and attempts). This is similar to trends observed before the COVID-19 pandemic (*7*). These findings are consistent with the trends of rising rates of suicide risk among females (*8*) and highlight the potential effect of the COVID-19 pandemic mitigation measures that might have increased students’ social isolation and anxiety, leading to the onset or exacerbation of adolescent mental health concerns and suicidal thoughts and behaviors (*9*).

The COVID-19 pandemic had a differential effect on suicide risk among male and female youths (*10*). Consistent with a study that cited a 50.6% increase in mean weekly emergency department visits during February and March 2021 for suspected suicide attempts among females aged 12–17 years versus 3.7% among males of the same age (*10*), the current study found significant increases in female students who reported seriously considered attempting suicide, making a suicide plan, and attempting suicide. In 2021, approximately one third of female students reported that they had seriously considered attempting suicide, approximately one fourth reported making a suicide plan, and 13.3% reported attempting suicide.

In 2021, 9th- and 10th- grade female students were significantly more likely than 12th-grade students to seriously consider attempting suicide, make a suicide plan and report a suicide attempt; 9th-grade female students compared with 12th-grade female students were more likely to have made a suicide attempt that required medical treatment. These findings concur with previous research indicating that both females and those in younger grade levels (7th and 9th grade) are more likely than males and those in older grade levels (11th and 12th grade) to report both nonfatal self-harm and suicide attempts (*11*). Because of the increased prevalence of suicidal thoughts and behaviors among female students, particularly for those in 9th and 10^th^ grade, the importance of early prevention and intervention to prevent suicide is evident. Given multiple developmental needs, determining how to best implement developmentally appropriate, evidence-based strategies to reach elementary and middle school-age youths might be a critical step in disrupting the upward trend of suicidal behaviors and might require further research on programs that are effective for young children and youths and their implementation.

In 2021, among both female and male students who reported having had same sex sexual contact or opposite sex sexual contact, the prevalence of all outcomes was significantly higher than students with no sexual contact. In addition, in 2021, prevalence of all four outcomes was found to be significantly higher among male LGBQ+ students compared with male heterosexual students. The prevalence of three outcomes (seriously consider suicide, plan and attempt) was higher among female LGBQ+ students compared with female heterosexual students. These findings are consistent with previous research that indicate that LGBQ+ youths are at increased risk for suicidal thoughts and behaviors (*6*) (https://www.cdc.gov/suicide/facts/disparities-in-suicide.html). Creating a safe and supportive school environment for LGBQ+ students by implementing gay and straight alliances, training teachers on LGBQ+ inclusivity, and using an LGBQ+ curriculum, has been associated with lower odds for suicide-related thoughts and behaviors among LGBQ+ students (https://www.liebertpub.com/doi/10.1089/lgbt.2021.0133). Creating affirming environments in both home and online spaces has also been determined to reduce suicide attempts among LGBQ+ youths (https://www.thetrevorproject.org/resources/article/facts-about-lgbtq-youth-suicide).

Significant prevalence increases among those who seriously considered attempting suicide, made a suicide plan, and reported making a suicide attempt were observed from 2019 to 2021 by race and ethnicity. For example, increases were noted between 2019 and 2021 among Black, Hispanic, and White female students who seriously considered attempting suicide, among White, and Hispanic female students who made a suicide plan, and among White female students who reported attempting suicide. A substantial number of students rely on school-based mental health care, especially youths in racial and ethnic minority groups from under-resourced families (*12*); the increased prevalence of suicidal behaviors among these students might reflect a lack of access to mental health care as schools closed to offset transmission of COVID-19.

Although lack of access to mental health services might have contributed to increased suicide risk, certain other factors, including substance misuse, family or relationship problems, community violence, and discrimination, might have also contributed to the increased risk (*13*). These factors highlight the need for a comprehensive approach to suicide prevention that is aimed at preventing suicide risk, supporting those at increased risk for suicide, preventing reattempts, and supporting survivors of suicide loss (*13*).

## Limitations

General limitations for the YRBS are available in the overview report of this supplement (*5*). The findings in this report are subject to at least two additional limitations. First, the 2021 national YRBS expanded its options to the sexual identity question to be more inclusive of how students self-identify; thus, the results from this question should not be compared to results from previous surveys. Second, this analysis was conducted among all students and did not stratify based on whether students had considered suicide; suicidal behaviors might differ between those who experienced suicidal thoughts and those who did not.

## Future Directions

There were marked differences in suicidal thoughts and behaviors by sexual orientation. Future research examining how intersectional identities and social norms regarding gender, race and ethnicity, and sexual orientation contribute to risk for suicidal behaviors can guide the development of effective interventions. Expanding the research evidence on the factors contributing to racial and ethnic differences in suicidal thoughts and behaviors can guide the development of inclusive intervention approaches. Increasing access to culturally and linguistically relevant mental health services can improve suicide prevention for racial and ethnic minority youths by connecting them to services that address their lived experiences (*14*). Better understanding how the pandemic exacerbated suicide risk could be important in developing school and community-based interventions for implementation during times of infrastructure disruption. These interventions should address common risk and protective factors considering student sex, grade, sexual identity, and race and ethnicity.

The CDC Suicide Prevention Resource for Action identifies strategies for a comprehensive approach to suicide prevention (*13*) that addresses the multiple factors associated with suicide risk. The implementation of school-based strategies, in addition to other community-based supports, has the potential for great reach and importance for youth suicide prevention. For example, creating safe and supportive environments for students by promoting school connectedness, teaching coping and problem solving, gatekeeper training, and implementing mental health services and programs can support youths in school (*13*,*15*).

## Conclusion

From 2019 to 2021, the prevalence of suicidal thoughts and behaviors increased among certain demographic groups but was stable among other groups. The increased prevalence of suicidal thoughts and behaviors among females, particularly among 9th- and 10th-grade females and Black, Hispanic, and White female students, as well as youths identifying as LGBQ+ and youths with same sex sexual contact, point to notable disparities warranting further consideration. Understanding the stable prevalence of suicidal thoughts and behaviors among males overall during a major infrastructure disruption (e.g., during the COVID-19 pandemic) could yield insights into protective factors. A combination of risk and protective factors at the individual, relationship, community, and societal levels likely contributes to the differences in suicidal thoughts and behaviors among sexual minority youths, different racial and ethnic groups and the differences observed by sex and grade. A comprehensive approach to suicide prevention, which reduces risk and supports youths at increased risk, provides support to those at risk and can ultimately save lives (*13*).

## Footnotes

* 45 C.F.R. part 46.102(l)(2), 21 C.F.R. part 56; 42 U.S.C. §241(d); 5 U.S.C. §552a; 44 U.S.C. §3501 et seq.
